# Tracking the evolution of alternatively spliced exons within the *Dscam *family

**DOI:** 10.1186/1471-2148-6-16

**Published:** 2006-02-16

**Authors:** Mack E Crayton, Bradford C Powell, Todd J Vision, Morgan C Giddings

**Affiliations:** 1 Department of Microbiology and Immunology, The University of North Carolina at Chapel Hill, Chapel Hill, NC 27599, USA; 2 Department of Biomedical Engineering, The University of North Carolina at Chapel Hill, Chapel Hill, NC 27599, USA; 3 Curriculum in Genetics and Molecular Biology, The University of North Carolina at Chapel Hill, Chapel Hill, NC 27599, USA; 4 Department of Biology, The University of North Carolina at Chapel Hill, Chapel Hill, NC 27599, USA; 5 Department of Biology, Xavier University of Louisiana, New Orleans, LA 70125, USA

## Abstract

**Background:**

The *Dscam *gene in the fruit fly, *Drosophila melanogaster*, contains twenty-four exons, four of which are composed of tandem arrays that each undergo mutually exclusive alternative splicing (4, 6, 9 and 17), potentially generating 38,016 protein isoforms. This degree of transcript diversity has not been found in mammalian homologs of *Dscam*. We examined the molecular evolution of exons within this gene family to locate the point of divergence for this alternative splicing pattern.

**Results:**

Using the fruit fly *Dscam *exons 4, 6, 9 and 17 as seed sequences, we iteratively searched sixteen genomes for homologs, and then performed phylogenetic analyses of the resulting sequences to examine their evolutionary history. We found homologs in the nematode, arthropod and vertebrate genomes, including homologs in several vertebrates where *Dscam *had not been previously annotated. Among these, only the arthropods contain homologs arranged in tandem arrays indicative of mutually exclusive splicing. We found no homologs to these exons within the *Arabidopsis*, yeast, tunicate or sea urchin genomes but homologs to several constitutive exons from fly *Dscam *were present within tunicate and sea urchin. Comparing the rate of turnover within the tandem arrays of the insect taxa (fruit fly, mosquito and honeybee), we found the variants within exons 4 and 17 are well conserved in number and spatial arrangement despite 248–283 million years of divergence. In contrast, the variants within exons 6 and 9 have undergone considerable turnover since these taxa diverged, as indicated by deeply branching taxon-specific lineages.

**Conclusion:**

Our results suggest that at least one *Dscam *exon array may be an ancient duplication that predates the divergence of deuterostomes from protostomes but that there is no evidence for the presence of arrays in the common ancestor of vertebrates. The different patterns of conservation and turnover among the *Dscam *exon arrays provide a striking example of how a gene can evolve in a modular fashion rather than as a single unit.

## Background

Many genes containing tandem exon arrays undergo mutually exclusive alternative splicing, in which only one exon variant within a tandem array of related variants is incorporated into the mature transcript [1,2]. In the fruit fly, *Drosophila melanogaster*, this form of alternative splicing can potentially produce 38,016 distinct, mature RNAs from a single gene, Down Syndrome Cell Adhesion Molecule (*Dscam*) [3-6]. Similar arrays of exon variants have been reported in homologs of *Dscam *from other Diptera (*D. pseudoobscura *and *D. virilis *and the mosquito *Anopheles gambiae*), the honeybee *Apis mellifera *(Hymenoptera) [4,7,8], and most recently the flour beetle *Tribolium castaneum *(Coleoptera) and the silk moth *Bombyx mori *(Lepidoptera) [9].

The fly *Dscam *gene encodes proteins that are members of the immunoglobulin (Ig) superfamily of cell adhesion molecules, and appears to be involved in neuronal axon guidance and bifurcation [4,10-12]. It may also be involved in adaptive immunity in insects [9]. The protein is comprised of both an extracellular domain that is highly conserved between insect and vertebrate species, and an intracellular domain for which there is little sequence similarity between the insects and vertebrates. Nonetheless, it has been shown in both groups that the intracellular domain activates Pak1 [13], though in the insects this is an indirect interaction through the adaptor protein Dock.

The name of the gene derives from the fact that one of the designated human *Dscam *homologs maps to a Down syndrome-associated region on chromosome 21; Down syndrome is the most common inherited form of mental retardation [14,15]. Another homolog, *Dscam-like*, maps to a locus on chromosome 11 that is associated with the neurological disorders Giles de la Tourette syndrome and Jacobsen syndrome [16]. Though evidence indicates that human *Dscam *may perform similar functions as its fly homolog, the specific functional role(s) of the human *Dscam *protein are still under investigation [13,14]. To date, the human *Dscam *gene has been reported to produce only three alternative transcripts [16], raising a question about the extent to which transcriptional diversity is found among members of the gene family as a whole.

To address this question, we first located then analyzed the evolution of exons homologous to those within fly *Dscam *that contain tandem arrays of alternative splice variants: exons 4 (12 variants), 6 (48 variants), 9 (33 variants), and 17 (2 variants) [4,5,17]. These exons code for extracellular and transmembrane domains of the protein. A tandem exon array consists of multiple variants, each of which contains a unique alternative 5' (donor) and 3' (acceptor) splice site. Only one variant from an array is incorporated into each mature transcript, and different transcripts may incorporate different variants. The variants in *Dscam *presumably arose through tandem exon duplication, since they share similar sequences. Exon arrays containing three or fewer variants have been observed in humans [18,19], and few cases of large tandem arrays are known, such as *Pcdh *genes, some of which have up to 22 variable exons in a tandem array [20]. Several other genes in vertebrates have also been found to have variable first exons in tandem arrays which undergo alternative splicing [21]. Notably, however, such exon arrays appear to be absent from human *Dscam *and *Dscam-like *genes [16].

Because there is a striking difference in the levels of transcript diversity that can be generated among the homologs of this gene [7,8,14], it is an intriguing system for studying the evolution of mutually exclusive splicing. Here we address a number of outstanding questions about this gene family. Do tandem arrays of exons homologous to those in fly *Dscam *exist elsewhere in non-insect genomes? If not, were tandem arrays lost along the lineage leading to mammals or gained along the lineage leading to insects? Do the exon variants undergo turnover within the array and, if so, how rapidly? Are the rate and mode of evolution similar among the different exon arrays?

**Figure 1:**
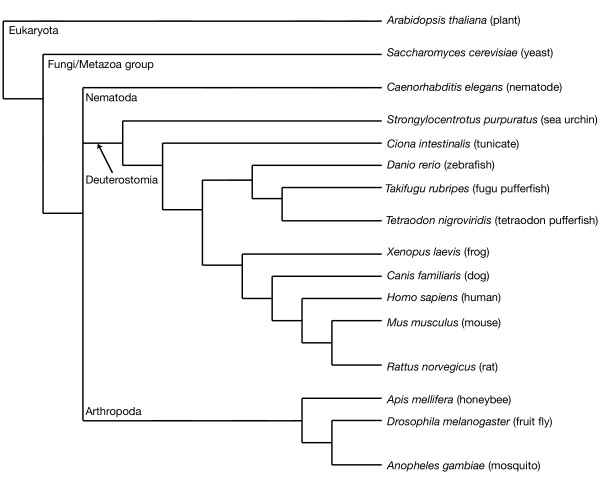
**Phylogenetic relationships among the organisms included in this study, after Hedges [26]**. Taxonomic categories mentioned in the text are those used by NCBI [42].

To answer these questions, we began with the *Dscam *exon variants from each of the fly exon arrays 4, 6, 9 and 17, performing searches for homologous exons amongst sixteen genomes (Figure 1) and filtering those matches by criteria described in the methods. Once the sets of homologous exons were identified, we used Bayesian methods to infer the phylogeny of each one.

## Results

### Homologous sequences are present only within the Arthropoda, Nematoda, and Vertebrata

The phylogenetic relationships among the sixteen taxa included in this study are shown in Figure 1. Collectively, we identified a total of 13,107 exon sequences homologous to the fly exon arrays in the vertebrate, nematode and insect genomes. Homologous sequences were not found in the yeast, sea urchin, tunicate, or plant genomes. Assuming that homologous sequences were most likely present in the most recent common ancestor of vertebrates, nematodes, and insects rather than acquired via horizontal transfer, the absence of homologous sequences from sea urchins and tunicates suggests that they have been lost from these genomes or have diverged beyond recognition. We also searched for sequences homologous to the constitutive exons that flank the tandem arrays (exons 3, 5, 8 and 10) within the fly *Dscam *gene. Homologs were not found in the yeast or plant genomes but were found in the tunicate, sea urchin, nematode, vertebrate, and insect genomes. The fact that regions similar to constitutive exons of *Dscam *were found within tunicate and sea urchin but that the alternatively spliced exons were not suggests unique selective pressures between the two classes of exon.

The number and taxonomic distribution of homologs varies among the exons. Sets seeded with exons 4, 6, 9 and 17 of *D. melanogaster *contained 84, 130, 12,515 and 378 members, respectively. Each set contained all of the exons in the corresponding *D. melanogaster *exon array. For exon arrays 4, 9 and 17, each mammalian species possessed only one homolog within an annotated *Dscam *gene (human *Dscam*: [GenBank:NM_001389]; mouse *Dscam*: [GenBank:AF315558]; and rat *Dscam*: [GenBank:NM_133587]). Each also possesses a single homolog to the *Dscam-like *gene (human *DscamL*: [GenBank:AF491813]; mouse *DscamL*: [GenBank:AF487345]; and rat *DscamL*: [GenBank:XM_236202]). Variants of the fly exon 9 array are also homologous to over 100 sequences within the *titin *gene on human chromosome 2. We have also found homologous sequences to exon arrays 4, 9 and 17 in zebrafish, tetraodon, fugu, frog, and dog genomes where neither *Dscam *nor *Dscam-like *had been annotated at the time of this study.

Each of the 130 sequences homologous to the exon 6 array were from the insect genomes studied (fly, mosquito or honeybee). There do not appear to be any sequences homologous to the exon 6 array within either of the mammalian paralogs, *Dscam *or *Dscam-like*, nor anywhere else within a non-insect genome.

Within the insect genomes we found the reported number of annotated splice variants for each exon array [4,5,17]. In fly we identified twelve exon 4 variants, forty-eight exon 6 variants, thirty-three exon 9 variants and two exon 17 variants. We also found the expected mosquito and honeybee exon 4 variants (numbering 14 and 8, respectively) and the exon 6 variants (numbering 30 and 45, respectively). Fly exon 9 variants are homologous to exon 10 variants in both mosquito and honeybee, where we found the reported thirty-eight exon 10 splice variants in mosquito and seventeen in honeybee. Fly exon 17 is homologous to mosquito exon 14 and honeybee exon 22. We found the two variants for each of these exons. Interestingly, we identified new homologs to fly exons 4 and 6 that were dispersed throughout the fly, mosquito and honeybee genomes. These are shown as "homolog_ (suffix)" in figures 3 and 6, and their chromosomal locations are listed in Additional file 5.

### Tandem arrays are only present in the insect *Dscam *genes

None of the vertebrate exon homologs we found, including those in genomes where *Dscam *was unannotated, were contained within tandem arrays, with the exception of matches in the human *titin *gene. The new vertebrate homologs appear to correspond to multiple genes in each vertebrate genome examined, as evidenced by their physical locations [see Additional file 5]. Furthermore, the newly identified homologs within fly, mosquito and honeybee were all found at unique positions, indicating that none of these are situated in tandem arrays. We examined the 183 homologs of fly exon 9 found within the human *titin *gene, by searching these sequences against *titin *transcripts. The transcript CAD12456.1 had matches for 158 of the fly homologs with 100% identity, and NP_596869.2 (*titin *variant N2A) had 156 perfect matches. The simultaneous matching of a majority of exons within individual transcripts strongly suggests that these homologs do not undergo mutually exclusive splicing. Our result does not rule out other forms of splicing, and in fact *titin *is known to undergo extensive splicing that results in variable length transcripts due to exon-skipping (e.g. [22-25]).

Taken in sum, these results indicate that none of these *Dscam *exon homologs undergo mutually exclusive splicing outside of the arthropods.

### Vertebrate *Dscam *homologs can be distinguished by core residues

We generated protein multiple sequence alignments (MSAs) for the four sets of homologous sequences. There are 47 residues in the edited alignment for exon 4, 27 for exon 6, 53 for exon 9, and 41 for exon 17. The alignments consist of homologs from the annotated *Dscam *genes and the new homologs we found.

**Figure 2:**
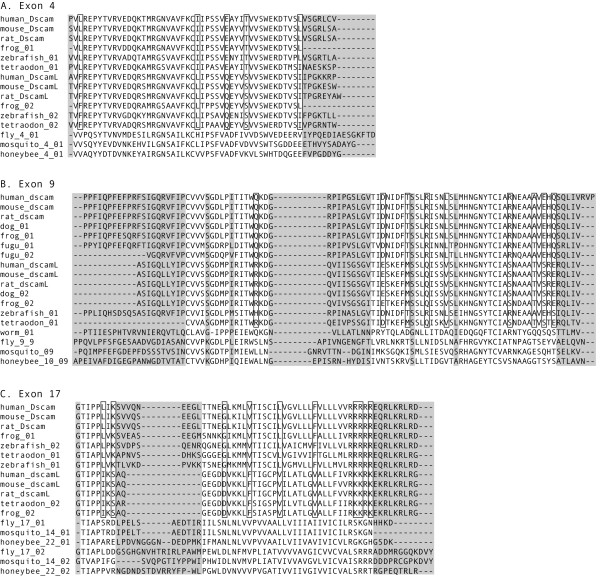
**Multiple sequence alignment for homologs to fly *Dscam *exons 4 (panel A), 9 (panel B), and 17 (panel C)**. Representative sequences from fly, mosquito and honeybee *Dscam *exon arrays are aligned with homologous sequences from eight vertebrate genomes. Shaded areas indicate columns whose residues produced gaps within the alignment and were excluded from further phylogenetic analyses as discussed in the Methods section. Boxed residues distinguish mammalian *Dscam *and *Dscam-like *sequences.

Analysis of the MSAs revealed that the sequences corresponding to the mammalian *Dscam *and *Dscam-like *genes (which contain homologs to fly exons 4, 9 and 17) could be distinguished using 5–10 specific residues from each exon. Panels A, B and C within Figure 2 show a portion of the MSA for fly exons 4, 6 and 9, respectively. The figure shows all of the vertebrate *Dscam *homologs and a single homolog from each of the three insects. The exon 4 MSA contains diagnostic residues at five positions: 2, 27, 33, 37 and 48 (boxed in Figure 2A): the *Dscam *motif for these six residues is LIETL while the *Dscam-like *motif is FLQSI. Within the exon 9 MSA the sequences corresponding to the mammalian *Dscam *and *Dscam-like *genes can be distinguished by residues at nine positions: 13, 18, 23, 26, 30, 42 47, 49 and 51 (boxed in Figure 2B): QDTRLRAEQ in *Dscam *and REMQVSTSE in *Dscam-like*. The exon 17 MSA contains diagnostic residues at nine different positions: 5, 7, 12, 17, 23, 30, 38, 39, and 41 (boxed in Figure 2C): LKGVLFRRR and ISDFVVKKK in *Dscam *and *Dscam-like*, respectively. These diagnostic residues can be used to infer whether unannotated sequences belong to the mammalian *Dscam *or *Dscam-like *lineages.

**Figure 3:**
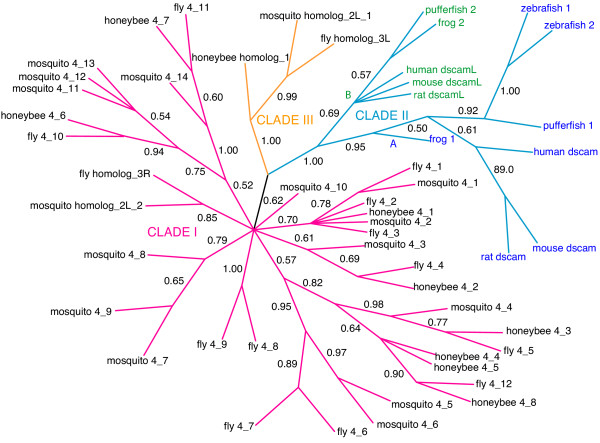
**Bayesian phylogeny of *Dscam *exon 4 homologs**. Only branches with posterior probabilities greater than 0.5 are shown (probabilities are shown beside each branch). Roman numerals (I, II and III) and colored branches denote the three major clades (magenta, orange and cyan, respectively). Subclades (A and B) of Clade III are denoted with colored text labels, blue (*Dscam*_suffix) and green (*DscamL*_suffix).

### Phylogenetic relationships

**Figure 4:**
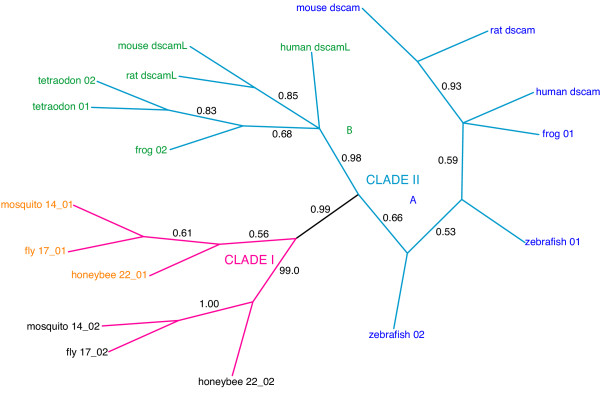
**Bayesian phylogeny of *Dscam *exon 17 homologs**. Only branches with posterior probabilities greater than 0.5 are shown (probabilities are shown beside each branch). Roman numerals (I and II) and colored branches denote the two major clades (magenta, and cyan, respectively). Subclades (A and B) of Clade I are denoted with colored text labels, orange (prefix_01) and black (prefix_02). Subclades (A and B) of Clade II are denoted with colored text labels, blue (*Dscam*_suffix) and green (*DscamL*_suffix).

**Figure 5:**
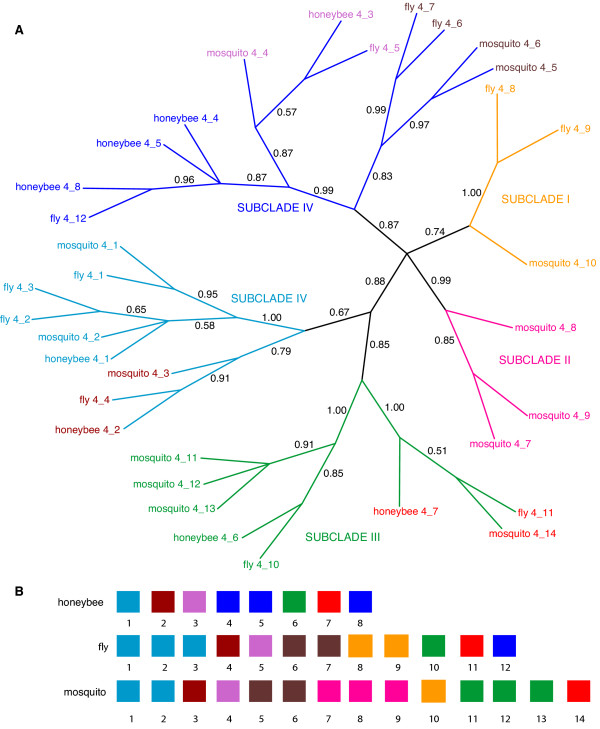
**Phylogeny of the annotated insect (fly, mosquito and honeybee) *Dscam *exon 4 homologs and organization of tandem arrays**. (A) Only branches with a posterior probability greater than 0.5 are shown (probabilities are shown beside each branch). Roman numerals (I thru V) and colored branches denote the five major subclades of the Clade I (Figure 3) sequences. Each major subclade contains at least one sequence from each fly, mosquito and honeybee. 5B. (B) Comparison between tandem arrays of honeybee (top), fly (middle) and mosquito (bottom) annotated *Dscam *exon 4 variants. Colored boxes represent the exon variants and the numbers below indicate the position of the variant within the tandem array. The box colors correspond to the colors of the text labels (rather than branch colors) shown in Figure 5A.

**Figure 6:**
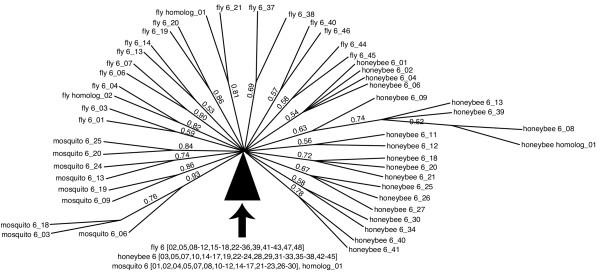
**Phylogeny of *Dscam *exon 6 homologs**. Only branches with posterior probabilities greater than 0.5 are shown (probabilities are shown beside each branch). The black pie-wedge represents a confluence of lineages whose branches all radiate from a common node and these branches remain unresolved.

**Figure 7:**
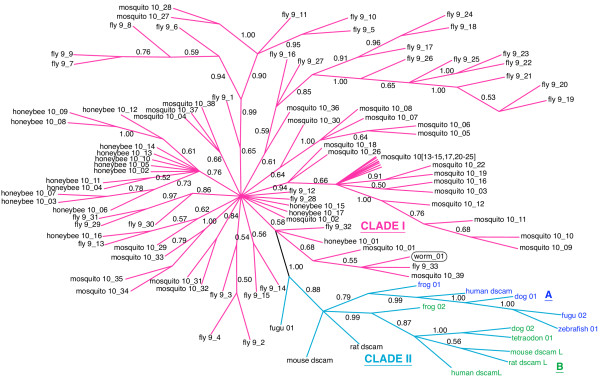
**Phylogeny of *Dscam *exon 9 homologs**. Only branches with posterior probabilities greater than 0.5 are shown (probabilities are shown beside each branch). Roman numerals (I and II) and colored branches denote the two major clades (magenta, and cyan, respectively). Subclades (A and B) of Clade II are denoted with colored text labels, blue (*Dscam*_suffix) and green (*DscamL*_suffix).

We obtained unrooted phylogenetic trees by Bayesian analysis of the nucleotide sequence alignments for each of the four exons. In each case, the position of the root is uncertain since there is no clear outgroup sequence. These trees are shown in Figs. 3, 4, 5, 6, 7 and discussed below.

### Tandem arrays for exons 4 and 17 are highly conserved among insects

Exons 4 and 17 share similar features, as shown in Figures 3 and 4, respectively. In both trees, there is a well-supported high-level clade (clade I – magenta) that includes the annotated *Dscam *exon variants from fly, mosquito and honeybee. The clade I sequences in the exon 4 tree also include a single unannotated sequence from fly and mosquito.

In both trees, Clade I can be subdivided into well-supported subclades, two in exon 17 (represented by orange and black text labels in Figure 4) and at least five subclades in exon 4, as shown in Figure 5A. Though the definition of the subclades is somewhat arbitrary, most of the subclades are represented by at least one variant in fly, mosquito and honeybee. The substantial depth of each subclade relative to the shallow divergence among sequences from fly, mosquito and honeybee suggests that the diversification of the variant lineages within the tandem array (and, by implication, the presence of mutually exclusive alternative splicing) long predates the divergence of these three species.

For exon 4, the position of variants from each subclade within the array is strikingly similar in the three species, as shown in Figure 5B. This indicates that there has been very little turnover of exon variants for at least 243–282 million years since these three species separated [26], and judging by the branch lengths, probably considerably longer. This conservation in the structure of the array strongly suggests that the subclades of alternatively spliced variants evolved specialized functions long prior to divergence of the insect taxa under study.

The trees for exons 4 and 17 also contain a well supported clade (clade II – cyan) that contains only vertebrate sequences. In both cases, Clade II can be subdivided into two subclades. Subclade A is comprised of sequences from the *Dscam *lineage (blue) and subclade B is comprised of sequences from the *Dscam-like *lineage (green). The vertebrate *Dscam *and *Dscam-like *sequences are more closely related to each other than either is to the insect *Dscam *genes.

Only the exon 4 tree contains Clade III (orange) and there is strong support (1.0 posterior probability) for the monophyly of this small clade of unannotated insect sequences. These three sequences lie outside the annotated *Dscam *exons in fly (fly homolog 01), mosquito (mosquito homolog 01) and honeybee (honeybee homolog 01). The branch lengths separating the annotated insect *Dscam *sequences (clade I) from clades II (vertebrate sequences) and clade III (unannotated insect sequences) are roughly comparable.

### Only insects contain homologs to exon 6

Exon 6 presents a strong contrast to exon 4 and 17. The phylogenetic tree for exon 6 is comprised of the annotated *Dscam *exon variants from fly, mosquito and honeybee, together with unannotated sequences from these same genomes (Figure 6). No homologous sequences were found within the vertebrates or other non-insect genomes. Many of the variant lineages radiate from near the midpoint of the tree and have relatively weak support. The clustering of variants from individual species suggests that many of the present-day variants proliferated after divergence of the insect species, or that the variants have undergone recombination.

### Exon 9 has experienced high turnover since divergence of the insects

Exon 9 is similar to exons 4 and 17 in that there is relatively good resolution of the phylogeny (Figure 7), and sequences from vertebrates are present. However, the turnover within the insects is, at least superficially, more similar to the pattern seen in exon 6. There are two well-supported high-level clades. Clade I (magenta) contains the annotated insect *Dscam *exon variants and also an unannotated sequence from the nematode, while clade II (cyan) contains the vertebrate sequences.

The monophyly of clade I is supported by a branch with a posterior probability of 0.99, assuming that the root is outside the group. The species-specific clusters of exon variants in Clade I indicate that, like exon 6, exon 9 has undergone substantial radiation after divergence of the insect species or that there has been recombination among the variants. The single nematode homolog (worm_01, circled in Fig. 7) is nested among insect sequences within Clade I. The position of the sequence within the clade suggests that it diverged from its closest insect homologs after the establishment of the tandem array. This would imply that the array was present but lost in the Nematoda (and possibly the Deuterostomia). However, the two branches supporting this derived position have relatively low support (0.68 and 0.55), and so the nematode sequence could plausibly branch from a node at the base of this clade. This latter placement would be consistent with the origin of the array after the divergence of the Arthropoda from the Nematoda (and Deuterostoma).

Clade II of exon 9, containing the vertebrate sequences, is supported by a posterior probability of 0.99. As in exon 4, the vertebrate *Dscam *and *Dscam-like *sequences are much more closely related to each other than either is to fly *Dscam*. The annotated mammalian *Dscam *sequences and *Dscam-like *sequences can also be divided into two well-supported subclades A and B (colored blue and green, respectively in Figure 7) with the exception of the two rodent *Dscam *sequences. Subclade A (blue) contains the annotated human *Dscam *sequence and single sequences from dog, fugu and zebrafish. Subclade B (green) contains the annotated human and rodent *Dscam-like *sequences, a second sequence from both frog and dog, and the sole sequence from tetraodon. There is strong support for the divergence of the single fugu sequence (fugu_01) prior to the split between subclades A and B. Curiously, the well-supported relationships within clade II are not entirely consistent with the phylogeny of the vertebrates. For example, clades containing dog and fish sequences are sister to the other mammalian sequences in both Subclades A and B. This suggests that either the phylogenetic topology is incorrect or that there has been a complex history of unobserved duplications and losses.

### Conservation of exon-to-protein domain correspondence between fly and human

**Figure 8:**
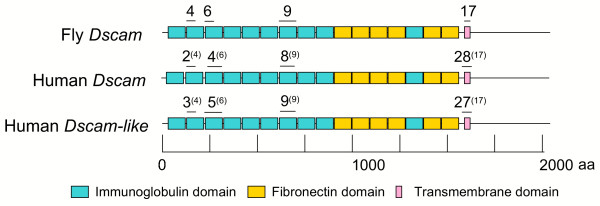
**Comparative protein domain structure of Human Dscam, Dscam-like and fly Dscam**. Based on results from Ensembl, UniProt, and InterProScan, the four exons in fly that undergo mutually-exclusive alternative splicing are marked above the protein subdomains (exons 4 and 6) or domains (exons 9 and 17) that they encode. The homologous exons in human *Dscam *and *Dscam-L *are marked above their corresponding domains in the encoded proteins, with the homologous fly exon for each in parentheses. The vertebrate homologs to fly exon 6 were located for the figure by their position in the global alignment, though the overall similarity was low for these compared to the other homologous exon pairs.

We identified the exons and corresponding putative protein domains for fly Dscam, Human Dscam, and Human Dscam-L, to examine the correspondence between the variable exons and domains. These results are shown in Figure 8. For the fly Dscam protein, InterProScan produced a structure very similar to that shown by Schmucker *et al*. [4], with nine consecutive Ig domains (SM00409) followed by six fibronectin domains (SM00060) with another Ig between the fourth and fifth fibronectin. Exons 4 and 6 correspond to the first part of Ig domains 2 and 3, respectively, and exon 9 covers all of Ig domain 7. This is followed by the transmembrane domain, corresponding to exon 17. Uniprot annotations for human Dscam and Dscam-L showed a highly similar domain structure to that obtained for fly. Interestingly, for each of the fly exons and their human homologs in both genes, the locations are nearly identical with respect to the protein domain structure.

## Discussion

The extraordinary difference between the fly and human *Dscam *homologs in the extent of potential transcript diversity motivated us to investigate the phylogenetic origin of mutually exclusive alternative splicing in the four exon arrays of the *Dscam *gene.

### The origins of mutually exclusive splicing in *Dscam*

Homologs to all four exons were present in tandem arrays in the insect genomes, as previously reported [4,7,8]. Three of the four exons (4, 9 and 17) also had homologs in vertebrates, and one (exon 9) had a homolog in nematode. However, outside of the three insects that we examined, no homologs were arranged in tandem arrays. This includes the nematode homolog to exon 9 and all the newly identified vertebrate sequences (from zebrafish, tetraodon, fugu, frog, and dog) from exons 4, 9 and 17. Thus, the absence of mutually exclusive alternative splicing in the human *Dscam *and *Dscam-like *genes [14,16,27] represents either an innovation in the lineage leading to insects, or one or more ancient losses from the common ancestor of insects, nematodes and vertebrates, approximately a billion years ago [26]. The one exon for which there is any evidence of loss in the lineage leading to vertebrates is exon 9, and this is due to a single worm sequence nested with low confidence within the clade of insect variants (Figure 7, clade I).

We found no homologs to the alternatively spliced fly *Dscam *exons within the yeast, tunicate, sea urchin and plant genomes. For the constitutive exons that we examined, no homologs were not found in yeast or plant genomes. However, potential homologs to several of the constitutive exons (3, 5, 8 and 10) were found within the tunicate and sea urchin genomes. Since the phylogeny indicates that homologs to the fly exon 9 group must have existed in the common ancestor of nematodes, deuterostomes and arthropods, any exon 9 homologs must have either been lost from the sea urchin and tunicate genomes or have diverged beyond recognition.

### Differential conservation of variants within tandem arrays in the insects

The sequences and numbers of exon variants among the insect *Dscam *tandem exon arrays are all surprisingly well conserved [8], though we found that the arrays have experienced very different patterns of proliferation and turnover. In exons 4 and 17, the variants were apparently established in an ancient burst of invention and have undergone relatively little turnover since divergence of the Diptera (fly and mosquito) and the Hymenoptera (honeybee). By contrast, exons 6 and 9 appear to have undergone substantial radiation even since the more recent divergence of fly and mosquito. Many nodes in the phylogenies of exons 6 and 9 have weak support, particularly for the basal branches. This suggests an early period of rapid radiation in these arrays. Alternatively, there may have been recombination among the variants early in the history of these two arrays. The contrast among the four different exons suggests that, as functional units within the *Dscam *gene, they are evolving largely independently of one another.

### Implications for the functional divergence of vertebrate and insect *Dscam *genes

The absence of tandem arrays in any of the vertebrate *Dscam *homologs indicates that they do not participate in mutually exclusive alternative splicing and therefore lack the diversity of protein isoforms generated through this form of splicing. We know that the intracellular domains of the insect and vertebrate proteins both participate in the same signaling pathway (Pak), but by different means [13]. As well, our results indicate strong conservation of the exon to protein domain correspondence between fly and the human Dscam homologs. The similarity in protein sequence of the insect and vertebrate extracellular Ig domains (three of which are encoded by the exon arrays), combined with recent experimental evidence for specific homophilic interactions [10,16] suggests that the encoded protein domains are functionally homologous. Furthermore, an Ig domain (homologous to fly exon 6) is absent from the human *Dscam *protein. These points raise the following question: is the functionality of the diverse *Dscam *protein isoforms in insects not necessary in the vertebrate homologs, or is it achieved through other means, possibly including other forms of post-transcriptional or post-translational processing?

The recent paper by Watson *et. al*, demonstrates that in the fruit fly and other insects, mutually exclusive alternative splicing in *Dscam *appears to play a role in adaptive immunity [9]. While this finding has led to the suggestion that mutually exclusive alternative splicing in the insect *Dscam *gene is solely related to its role in immunity, and not in axon guidance [28], there is a growing body of evidence that the alternative isoforms do in fact play a role in axon guidance [10,12,13]. Members of the immunoglobulin family in vertebrates are also involved in adaptive immunity, but generate protein isoform diversity through somatic gene rearrangements rather than alternative splicing at the RNA level. Since *Dscam *contains immunoglobulin domains, and is now shown to be involved in adaptive immunity in insects, there is an intriguing possibility that, in vertebrates, a diversity of protein isoforms is achieved through somatic rearrangement of the *Dscam *and *Dscam-like *genes.

### Other cases of mutually exclusive alternative splicing

Though tandem arrays of *Dscam *homologs are absent outside the insects, many other genes exhibiting alternative splicing have been found [1,2]. For example, in humans the *neurexin *and *titin *genes have been shown to participate in mutually exclusive splicing, [6,18,29] and the three *neurexin *genes can potentially generate more than one thousand isoforms through this process [6,18,30]. This indicates that mutually exclusive alternative splicing can generate diverse protein isoforms in humans as it does within insects. However, the mutually exclusive splicing in these genes involves tandem arrays of three or fewer variants. Where larger arrays of exons undergoing this type of splicing have been found in vertebrates, they appear to be confined to the first exons of the gene [21].

## Conclusion

Our findings illustrate a striking contrast between the conservation of several large exon arrays in insect *Dscam *and the lack of any such arrays among vertebrates. Contrary to previous reports of high turnover [8], we found evidence for the maintenance of ancient structural patterns within the arrays, especially within exon 4. The four tandem arrays show different patterns of proliferation and conservation but the weight of evidence points to all four arrays having evolved after the divergence of arthropods from deuterostomes and nematodes. It remains to be determined whether the contrast between insect and vertebrate *Dscam *reflects functional divergence between the genes, or whether a distinct mechanism is acting to produce a similar diversity of transcripts in vertebrates.

## Methods

### Sources of data

Genomes from sixteen species were used in our analyses. These included all species that were available within Ensembl version 20 [7,31]: *Homo sapiens *(human), *Mus musculus *(mouse), *Rattus norvegicus *(rat), *Danio rerio *(zebrafish), *Tetraodon nigroviridis *(tetraodon pufferfish), Takifugu rubripes (fugu pufferfish), Xenopus tropicalis (frog), *Canis familiaris *(dog), *Drosophila melanogaster *(fruit fly), *Anopheles gambiae *(mosquito), *Apis mellifera *(honeybee), *Caenorhabditis elegans *(nematode), *Saccharomyces cerevisiae *(yeast) and *Arabidopsis thaliana *(plant). We supplemented these with genome data from the tunicate, *Ciona intestinalis *[32], and the sea urchin, *Strongylocentrotus purpuratus *[8], two metazoan species that diverged intermediate to the divergence of arthropods and vertebrates, as this proved to be a critical phylogenetic junction in our initial analysis. We did not include any of the vertebrate genomes that have since been released (e.g. chicken, gray short-tailed opossum), as our results suggested that these would not be informative about the gain or loss of tandem exon arrays in *Dscam*. Figure 1 shows the phylogenetic relationships among the taxa included in this study [26].

### Identification of homologous exons

We searched genomic sequences as opposed to annotated gene or protein databases, for two reasons. First, this allowed us to identify both annotated and unannotated exons. Second, since protein databases usually contain only a single isoform for each protein [2,3], they do not allow direct identification of tandem arrays of alternatively spliced exons. These searches were performed using two different methods: an iterative BLAST and PSI-BLAST.

The first search method entailed iterative searches of a database containing all the genomes using tBLASTn [33]. The fly *Dscam *exon splice variants 4.1, 6.11, 9.9 and 17.1 were used to seed separate searches. These variants are the representative exons contained within the fly *Dscam *isoform as annotated in Genbank entry AF260530, which serves as our primary data source. Hits with E-values less than or equal to 0.01 were used as query sequences in successive rounds of BLAST. This process was iterated until no new hits were obtained.

The second method entailed an initial iterated PSI-BLAST [34] search against the NCBI nonredundant protein database to construct a position-specific scoring matrix for each exon variant. The PSI-BLAST searches were run for a maximum of 20 iterations using a BLOSUM62 transition weight matrix and the default gap opening and extension penalties of 11 and 1, respectively. Hits with E-values of less than or equal to 0.002 were included in subsequent iterations. The resulting position-specific scoring matrices were then used to perform PSI-TBLASTN searches against each of the sixteen genomes under study using the same search parameters as for the PSI-BLAST searches. All searches were performed with version 2.2.10 of the NCBI BLAST tools. Search results from the different variants of each exon were pooled and overlapping sequences were merged.

There were no substantive differences between the results obtained using the two methods. Those from the PSI-BLAST method are reported here unless otherwise indicated.

### Alignment and phylogenetic analysis

Protein multiple sequence alignments (MSAs) were constructed using ClustalW [32] and edited manually. Sequences were included in the analysis only if they created internal gaps of fewer than 10 consecutive residues when aligned with the annotated *Dscam *sequences. Only the most conserved regions were included the final alignments, and columns containing gaps were removed. For the protein alignments used with exons 4, 6, 9 and 17, see Additional files 1, 2, 3 and 4, respectively. See Additional file 5 for the genomic locations of the exons used in the alignment. The corresponding nucleotide alignments were generated using the protein alignments as guides. Phylogenetic trees were inferred using Mr. Bayes version 3.0 [35]. Nucleotides were partitioned into three sets based on their position within the codon. Within each set, sites were permitted to have independently varying rates according to a discrete gamma distribution with four states. Five independent Markov chains were initialized with a random tree and run for 500,000 iterations, with trees sampled every 100 iterations. Due to a longer convergence time, the exon 9 tree was run for one million iterations with sampling every 300 iterations. The first 100 sampled trees (10,000 iterations) for exons 4, 6 and 17 and the first 133 sampled trees (39,900 iterations) for exon 9 were discarded as "burn-in". The final trees included only branches with posterior probability greater than 0.5.

### Domain/Exon correspondence analysis

We performed a comparison of the putative protein domain locations versus exon locations for each of the human homologs to fly 4, 6, 9, and 17, as shown in Figure 8. [see Additional files 6 and 7 for the alignments].

The Human Dscam and Dscam-L domains were obtained from UniProt entries O60469 and Q8TD84, respectively [36,37], and the exon locations were obtained from the Ensembl annotation of proteins ENSP00000302472 (Dscam) and ENSG00000177103 (Dscam-L) [38]. The putative domain structure for fly was obtained by combining an InterProScan [39,40] on the translated *Dscam *sequence (CG17800-PA) with the domain structures reported by Schmucker *et al*. and Watson *et al*. [4,9]. We also performed a Smith-Waterman alignment of fly Dscam versus Human Dscam using EMBOSS (Gap open = 10.0, extension = 0.5) [41], and added annotation corresponding to the locations of these exons and putative domains [see Additional file 6]. The same was done for a comparison of fly Dscam versus Human Dscam-L [see Additional file 7].

## Authors' contributions

MEC constructed the alignments and phylogenetic trees and drafted the manuscript. BCP wrote the software to automate the sequence searches. MCG conceived of and coordinated the study. All four authors participated in the design of the study and interpretation of the results, and all authors read and approved the final manuscript.

## Supplementary Material

Additional File 1 **Alignment of *Dscam *exon 4 homologs**. Alignment of sequences found using variants of *Dscam *exon 4 from *Drosophila *as queries. ClustalW format, viewable as plain text. Click here for file

Additional File 2 **Alignment of *Dscam *exon homologs**. Alignment of sequences found using variants of *Dscam *exon 6 from *Drosophila *as queries. ClustalW format, viewable as plain text. Click here for file

Additional File 3 **Alignment of *Dscam *exon 9 homologs**. Alignment of sequences found using variants of *Dscam *exon 9 from *Drosophila *as queries. ClustalW format, viewable as plain text. Click here for file

Additional File 4 **Alignment of *Dscam *exon 17 homologs**. Alignment of sequences found using variants of *Dscam *exon 17 from *Drosophila *as queries. ClustalW format, viewable as plain text. Click here for file

Additional File 5 **Locations of sequences similar to *Dscam *exons**. Locations of the sequences referenced in additional files 1, 2, 3, 4 and in the text. Locations are given on chromosomal assemblies from Ensembl version 20 or from contigs referenced in the methods. The file is in Microsoft Excel format. Stop coordinates less than start coordinates indicate that elements are in the (-) strand. Click here for file

Additional File 6 A Smith-Waterman alignment of fly Dscam versus Human Dscam, with the sequences corresponding to exons highlighted by color (color legend in the file), and putative corresponding domain locations underlined. Click here for file

Additional File 7 A Smith-Waterman alignment of fly Dscam versus Human Dscam-L, with the sequences corresponding to exons highlighted by color (color legend in the file), and putative corresponding domain locations underlined. Click here for file

## References

[B1] Kondrashov F, Koonin E (2001). Origin of alternative splicing by tandem exon duplication. Human Molecular Genetics.

[B2] Letunic I, Copley R, Bork P (2002). Common exon duplication in animals and its role in alternative splicing. Human Molecular Genetics.

[B3] Graveley B (2001). Alternative splicing: increasing diversity in the proteomic world. Trends in Genetics.

[B4] Schmucker D, Clemens J, Shu H, Worby C, Xiao J, Muda M, Dixon J, Zipursky S (2000). Drosophila Dscam is an axon guidance receptor exhibiting extraordinary molecular diversity. Cell.

[B5] Black D (2000). Protein diversity from alternative splicing: A challenge for bioinformatics and post-genome biology. Cell.

[B6] Missler M, Sudhof TC (1998). Neurexins: three genes and 1001 products. Trends Genet.

[B7] Zdobnov E, von Mering C, Letunic I, Torrents D, Suyama M, Copley R, Christophides G, Thomasova D, Holt R, Subramanian G, Mueller HM, Dimopoulos G, Law J, Wells M, Birney E, Charlab R, Halpern A, Kokoza E, Kraft C, Lai Z, Lewis S, Louis C, Barillas-Mury C, Nusskern D, Rubin G, Salzberg S, Sutton G, Topalis P, Wides R, Wincker P, Yandell M, Collins F, Ribeiro J, Gelbart W, Kafatos F, Bork P (2002). Comparative genome and proteome analysis of Anopheles gambiae and Drosophila melanogaster. Science.

[B8] Graveley B, Kaur A, Gunning D, Zipursky SL, Rowen L, Clemens JC (2004). The organization and evolution of the Dipteran and Hymenopteran Down syndrome cell adhesion molecule (Dscam) gene. RNA.

[B9] Watson FL, Puttman-Holgado R, Franziska T, Lamar DL, Hughes M, Kondo M, Rebel V, Schmucker D (2005). Extensive Diversity of Ig-Superfamily Proteins in the Immune System of Insects. Science.

[B10] Wojtowicz WM, Flanagan JJ, Millard SS, Zipursky SL, Clemens JC (2004). Alternative splicing of Drosophila Dscam generates axon guidance receptors that exhibit isoform-specific homophilic binding. Cell.

[B11] Celotto A, Graveley B (2001). Alternative splicing of the Drosophila Dscam pre-mRNA is both temporally and spatially regulated. Genetics.

[B12] Zhan XL, Clemens JC, Neves G, Hattori D, Flanagan JJ, Hummel T, Vasconcelos ML, Chess A, Zipursky SL (2004). Analysis of Dscam diversity in regulating axon guidance in Drosophila mushroom bodies. Neuron.

[B13] Li W, Guan KL (2004). The Down Syndrome Cell Adhesion Molecule (DSCAM) interacts with and activates Pak. The Journal of Biological Chemistry.

[B14] Yamakawat K, Huot YK, Haendelt M, Hubert R, Chen XN, Lyons G, Korenberg J (1998). DSCAM: A novel member of the immunoglobulin superfamily maps in a Down syndrome region and is involved in the development of the nervous system. Human Molecular Genetics.

[B15] Epstein CJ (1986). Developmental genetics. Experientia.

[B16] Agarwala K, Ganesh S, Tsutsumi Y, Suzuki T, Amano K, Yamakawat K (2001). Cloning and functional characterization of DSCAML1, a novel DSCAM-like cell adhesion molecule that mediates homophilic intercellular adhesion. Biochemical and Biophysical Research Communications.

[B17] Ullrich B, Ushkaryov YA, Sudhof TC (1995). Cartography of neurexins: more than 1000 isoforms generated by alternative splicing and expressed in distinct subsets of neurons.. Neuron.

[B18] Ushkaryov YA, Petrenko AG, Geppert M, Sudhof TC (1992). Neurexins: Synaptic cell surface proteins related to the alpha-latrotoxin receptor and laminin. Science.

[B19] Wu Q (2005). Comparative genomics and diversifying selection of the clustered vertebrate protocadherin genes. Genetics.

[B20] Zhang T, Haws P, Wu Q (2004). Multiple variable first exons: a mechanism for cell- and tissue-specific gene regulation. Genome Res.

[B21] Neagoe C, Opitz CA, Makarenko I, Linke WA (2003). Gigantic variety: expression patterns of titin isoforms in striated muscles and consequences for myofibrillar passive stiffness. J Muscle Res Cell Motil.

[B22] Warren CM, Krzesinski PR, Campbell KS, Moss RL, Greaser ML (2004). Titin isoform changes in rat myocardium during development. Mech Dev.

[B23] Kolmerer B, Olivieri N, Witt CC, Herrmann BG, Labeit S (1996). Genomic organization of M line titin and its tissue-specific expression in two distinct isoforms. J Mol Biol.

[B24] Freiburg A, Trombitas K, Hell W, Cazorla O, Fougerousse F, Centner T, Kolmerer B, Witt C, Beckmann JS, Gregorio CC, Granzier H, Labeit S (2000). Series of exon-skipping events in the elastic spring region of titin as the structural basis for myofibrillar elastic diversity. Circ Res.

[B25] Hedges SB (2002). The origin and evolution of model organisms. Nature Reviews Genetics.

[B26] Barlow G, Micales B, Chen XN, Lyons G, Korenberg J (2002). Mammalian DSCAMs: roles in the development of the spinal cord, cortex, and cerebellum?. Biochemical and Biophysical Research Communications.

[B27] Du Pasquier L (2005). Immunology. Insects diversify one molecule to serve two systems. Science.

[B28] Bang ML, Centner T, Fornoff F, Geach AJ, Gotthardt M, McNabb M, Witt CC, Labeit D, Gregorio CC, Granzier H, Labeit S (2001). The complete gene sequence of titin, expression of an unusual approximately 700-kDa titin isoform, and its interaction with obscurin identify a novel Z-line to I-ban linking system.. Circ Res.

[B29] Occhi G, Rampazzo A, Beffagna G, Danieli GA (2002). Identification and characterization of heart-specific splicing of human neurexin 3 mRNA (NRXN3). Biochemical and Biophysical Research Communications.

[B30] Birney E, Andrews TD, Bevan P, Caccamo M, Chen Y, Clarke L, Coates G, Cuff J, Curwen V, Cutts T, Down T, Eyras E, Fernandez-Suarez XM, Gane P, Gibbins B, Gilbert J, Hammond M, Hotz HR, Iyer V, Jekosch K, Kahari A, Kasprzyk A, Keefe D, Keenan S, Lehvaslaiho H, McVicker G, Melsopp C, Meidl P, Mongin E, Pettett R, Potter S, Proctor G, Rae M, Searle S, Slater G, Smedley D, Smith J, Spooner W, Stabenau A, Stalker J, Storey R, Ureta-Vidal A, Woodwark KC, Cameron G, Durbin R, Cox A, Hubbard T, Clamp M (2004). An overview of Ensembl. Genome Res.

[B31] Thompson JD, Higgins DG, Gibson TJ (1994). CLUSTALW: improving the sensitivity of progressive multiple sequence alignment through sequence weighting, position-specific gap penalties and weight matrix choice.. Nucleic Acids Res.

[B32] Altschul SF, Gish W, Miller W, Myers EW, Lipman DJ (1990). Basic local alignment search tool.. J Mol Biol.

[B33] Altschul SF, Madden TL, Schaffer AA, Zhang J, Zhang Z, Miller W, Lipman DJ (1997). Gapped BLAST and PSI-BLAST: a new generation of protein database search programs. Nucleic Acids Res.

[B34] Ronquist F, Huelsenbeck J (2003). MrBayes 3: Bayesian phylogenetic inference under mixed models.. Bioinformatics.

[B35] Bairoch A, Apweiler R, Wu CH, Barker WC, Boeckmann B, Ferro S, Gasteiger E, Huang H, Lopez R, Magrane M, Martin MJ, Natale DA, O'Donovan C, Redaschi N, Yeh LS (2005). The Universal Protein Resource (UniProt). Nucleic Acids Res.

[B36] Boeckmann B, Bairoch A, Apweiler R, Blatter MC, Estreicher A, Gasteiger E, Martin MJ, Michoud K, O'Donovan C, Phan I, Pilbout S, Schneider M (2003). The SWISS-PROT protein knowledgebase and its supplement TrEMBL in 2003. Nucleic Acids Res.

[B37] Hubbard T, Barker D, Birney E, Cameron G, Chen Y, Clark L, Cox T, Cuff J, Curwen V, Down T, Durbin R, Eyras E, Gilbert J, Hammond M, Huminiecki L, Kasprzyk A, Lehvaslaiho H, Lijnzaad P, Melsopp C, Mongin E, Pettett R, Pocock M, Potter S, Rust A, Schmidt E, Searle S, Slater G, Smith J, Spooner W, Stabenau A, Stalker J, Stupka E, Ureta-Vidal A, Vastrik I, Clamp M (2002). The Ensembl genome database project. Nucleic Acids Res.

[B38] Mulder NJ, Apweiler R, Attwood TK, Bairoch A, Barrell D, Bateman A, Binns D, Biswas M, Bradley P, Bork P, Bucher P, Copley RR, Courcelle E, Das U, Durbin R, Falquet L, Fleischmann W, Griffiths-Jones S, Haft D, Harte N, Hulo N, Kahn D, Kanapin A, Krestyaninova M, Lopez R, Letunic I, Lonsdale D, Silventoinen V, Orchard SE, Pagni M, Peyruc D, Ponting CP, Selengut JD, Servant F, Sigrist CJ, Vaughan R, Zdobnov EM (2003). The InterPro Database, 2003 brings increased coverage and new features. Nucleic Acids Res.

[B39] Mulder NJ, Apweiler R, Attwood TK, Bairoch A, Bateman A, Binns D, Biswas M, Bradley P, Bork P, Bucher P, Copley R, Courcelle E, Durbin R, Falquet L, Fleischmann W, Gouzy J, Griffith-Jones S, Haft D, Hermjakob H, Hulo N, Kahn D, Kanapin A, Krestyaninova M, Lopez R, Letunic I, Orchard S, Pagni M, Peyruc D, Ponting CP, Servant F, Sigrist CJ (2002). InterPro: an integrated documentation resource for protein families, domains and functional sites. Brief Bioinform.

[B40] Rice P, Longden I, Bleasby A (2000). EMBOSS: The European Molecular Biology Open Software Suite. Trends in Genetics.

[B41] NCBI The NCBI taxonomy database. http://www.ncbi.nlm.nih.gov/entrez/query.fcgi?db=Taxonomy.

