# Supplementary material for: Tracking the evolution of alternatively spliced exons within the Dscam family
Source: BMC Evol Biol. 2006 Feb 16;6:16. doi: 10.1186/1471-2148-6-16 (PMC1397879; doi:10.1186/1471-2148-6-16)
Supplement: Additional File 6 — A Smith-Waterman alignment of fly Dscam versus Human Dscam, with the sequences corresponding to exons highlighted by color (color legend in the file), and putative corresponding domain locations underlined. [file 1471-2148-6-16-S6.pdf]

```
########################################
# Program: water
# Rundate: Sun Nov 18 2005 18:02:38
# Align_format: srspair
# Report_file: fly.water
########################################

#=======================================
#
# Aligned_sequences: 2
# 1: Fly
# 2: Human
# Matrix: EBLOSUM62
# Gap_penalty: 10.0
# Extend_penalty: 0.5
#
# Length: 2031
# Identity:     612/2031 (30.1%)
# Similarity:   965/2031 (47.5%)
# Gaps:         180/2031 ( 8.9%)
# Score: 2497.0
#
#
#=======================================
```

fly exon 4 and human Dscam homolog (exon 2), with Uniprot predicted Ig-like C2-type 1 underlined, and InterProScan predicted Fly IG PF00047.14 domain underlined

fly exon 6 and human homologous region (exon 4), with Uniprot predicted Ig-like C2-type 2 and InterProScan fly predicted IG PF00047.14 underlined

fly exon 9 and human homolog (exon 8), with Uniprot predicted Ig-like C2-type 1 underlined, and InterProScan fly predicted IG PF00047.14 underlined

fly exon 17 and human homolog (exon 26), with Human Dscam Uniprot predicted transmembrane domain underlined, and InterProScan fly predicted transmembrane domain underlined

```
Fly               41 FLKEPTNRIDFSNSTGAEIECKASGNPMPEIIWIRSDGTAVGDVPGLRQI     90
                     |:......:.|:::||..:.|.|:|.|...:.|..:.|..:.||||:|.:
Human             12 FVNASLQEVVFASTTGTLVPCPAAGIPPVTLRWYLATGEEIYDVPGIRHV     61

Fly               91 SSDGKLVFPPFRAEDYRQEVHAQVYACLARNQFGSIISRDVHVRAVVAQY    140
                     ..:|.|...||....:...:|...|.|.|.|..|.|.|:|||::||:.:.
Human             62 HPNGTLQIFPFPPSSFSTLIHDNTYYCTAENPSGKIRSQDVHIKAVLREP    111

Fly              141 YEADVNKEHVIRGNSAVIKCLIPSFVADFVEVVSWHTDEEENYFPGAEYD    190
                     |...|..:..:|||.||.||:|||.|..::.||||..| ..:...|:
Human            112 YTVRVEDQKTMRGNVAVFKCIIPSSVEAYITVVSWEKD-TVSLVSGS---    157
```

```
Fly          191 GKYLVLPSGELHIREVGPEDGYKSYQCRTKHRLTGETRLSATKGRLVITE   240
                  ::|:..:|.|:|::|..|||..:|:|.|:||.|||||.|  ...||.:::
Human        158 -RFLITSTGALYIKDVQNEDGLYNYRCITRHRYTGETRQS-NSARLFVSD   205

Fly          241 PVSSSPPKINALTYKPNIVESMAS--TAILCPAQGYPAPSFRWYKFIEGT   288
                 |.:|:|..::...::    ::||.  ..:.|.|.|:|.|.:||.|
Human        206 PANSAPSILDGFDHR----KAMAGQRVELPCKALGHPEPDYRWLK-----   246

Fly          289 TRKQAVVLNDRVKQVSGTLIIKDAVVEDSGKYLCVVNNSVGGESVETVLT   338
                  ....:.|:.|.::....|:|::....|||.|:|.|:|..|...|...|.
Human        247 -DNMPLELSGRFQKTVTGLLIENIRPSDSGSYVCEVSNRYGTAKVIGRLY   295

Fly          339 VTAPLSAKIDPPTQTVDFGRPAVFTCQYTGNPIKTVSWMKDGKAIGHSEP   388
                 |..||.|.|.|.......|.....:|..||...:.:||.::|:.:...:.
Human        296 VKQPLKATISPRKVKSSVGSQVSLSCSVTGTEDQELSWYRNGEILNPGKN   345

Fly          389 V---------LRIESVKKEDKGMYQCFVRNDQESAEASAELKLGGRFDPP   429
                 |         |.::.:.|.|.|.||||||.|:.||:...::.|  ....|
Human        346 VRITGINHENLIMDHMVKSDGGAYQCFVRKDKLSAQDYVQVVL--EDGTP   393

Fly          430 VIRQAFQEETMEPGPSVFLKCVAGGNPTPEISWELDGKKIANNDRYQVGQ   479
                 .|..||.|:.:.|...|.|.|...|.|.|.|:|.||...|.....:::.|
Human        394 KIISAFSEKVVSPAEPVSLMCNVKGTPLPTITWTLDDDPILKGGSHRISQ   443

Fly          480 YVTVNGDVVSYLNITSVHANDGGLYKCIAKSKVGVAEHSAKLNVYGLPYI   529
                 .:|..|:||||||||:|....|||:|:|.|.:..||..:.|::||.|...|
Human        444 MITSEGNVVSYLNISSSQVRDGGVYRCTANNSAGVVLYQARINVRGPASI   493

Fly          530 RQMEKKAIVAGETLIVTCPVAGYPIDSIVWERDNRALPINRKQKVFP-NG   578
                 |.|:....:||....:.|.|.|.|||..||.|.:::..||.|.:|..|. ||
Human        494 RPMKNITAIAGRDTYIHCRVIGYPYYSIKWYKNSNLLPFNHRQVAFENNG   543

Fly          579 TLIIENVERNSDQATYTCVAKNQEGYSARGSLEVQVMVPPQVLPFSFGES   628
                 ||.:.:|::..|:..|||....|...|...|:.|.|.|||.:.||.|...
Human        544 TLKLSDVQKEVDEGEYTCNVLVQPQLSTSQSVHVTVKVPPFIQPFEFPRF   593

Fly          629 AADVGDIASANCVVPKGDLPLEIRWSLNSAPIVNGENGFTLVRLNKRTSL   678
                 :  :|......|||..||||:.|.|..:..||  .|..|.|:..:: .||.
Human        594 S--IGQRVFIPCVVVSGDLPITITWQKDGRPI-PGSLGVTIDNID-FTSS   639

Fly          679 LNIDSLNAFHRGVYKCIATNPAGTSEYVAELQVNVPPRWILEPTDKAFAQ   728
                 |.|.:|:..|.|.|.|||.|.|...|:.::|.|.|.|||:::::|.|:....
Human        640 LRISNLSLMHNGNYTCIARNEAAAVEHQSQLIVRVPPKFVVQPRDQDGIY   689

Fly          729 GSDAKVECKADGFPKPQVTWKKAVGDTPGEYKDLKKSDNIRV-EEGTLHV   777
                 |....:.|.|:|:|.|.:.||.:.|....:::.:.:..:|:|  ..|:|.:
Human        690 GKAVILNCSAEGYPVPTIVWKFSKGAGVPQFQPIALNGRIQVLSNGSLLI   739
```

```
Fly            778 DNIQKTNEGYYLCEAINGIGSGLSAVIMISVQAPPEFTEKLRNQTARRGE  827
                   .::.:.:.|||||:..|.:|:.:|..:.::|:.|...|.......|.:|:
Human          740 KHVVEEDSGYYLCKVSNDVGADVSKSMYLTVKIPAMITSYPNTTLATQGQ  789

Fly            828 PAVLQCEAKGEKPIGILWNMNNMRLDPKNDNRYTIREEILSTGVMSSLSI  877
                   ...:.|.|.|||||.:.|...:..::|:. .||.:..:.:...|:|:|.|
Human          790 KKEMSCTAHGEKPIIVRWEKEDRIINPEM-ARYLVSTKEVGEEVISTLQI  838

Fly            878 KRTERSDSALFTCVATNAFGSDDASINMIVQEVPEMPYALKVLDKSGRSV  927
                   ..|.|.||..|:|.|.|::|.|...|.:.|||.|:.| .:::.|...|::
Human          839 LPTVREDSGFFSCHAINSYGEDRGIIQLTVQEPPDPP-EIEIKDVKARTI  887

Fly            928 QLSWAQPYDGNSPLDRYIIEFKRSRASWSEIDRV-IVPGHTTEAQVQKLS  976
                   .|.|...:|||||:..|.||.|....||....|. .|......|.:..:.
Human          888 TLRWTMGFDGNSPITGYDIECKNKSDSWDSAQRTKDVSPQLNSATIIDIH  937

Fly            977 PATTYNIRIVAENAIGTSQSSEAVTIITAEEAPSGKPQNIKVEPVNQTTM 1026
                   |::||:||:.|:|.||.|:.|..:||...|.||.|.||.:.:||::..::
Human          938 PSSTYSIRMYAKNRIGKSEPSNELTITADEAAPDGPPQEVHLEPISSQSI  987

Fly           1027 RVTWKPPPRTEWNGEILGYYVGYKLSNTNSSYVFETINFITEEGKEHNLE 1076
                   |||||.|.:...||.|.||.:||:..:|..::.|..|: :...|......
Human          988 RVTWKAPKKHLQNGIIRGYQIGYREYSTGGNFQFNIIS-VDTSGDSEVYT 1036

Fly           1077 LQNLRVYTQYSVVIQAFNKIGAGPLSEEEKQFTAEGTPSQPPSDTACTTL 1126
                   |.||..:|||.:|:||.|:.|.|||.|:|....|.|..||.||.:......
Human         1037 LDNLNKFTQYGLVVQACNRAGTGPSSQEIITTTLEDVPSYPPENVQAIAT 1086

Fly           1127 TSQTIRVGWVSPPLESANGVIKTYKVVYAPS--DEWYDETKRHYKKTASS 1174
                   :.::|.:.|.:...|:.||:::.::|:|..:  |....|.|.  ..|...
Human         1087 SPESISISWSTLSKEALNGILQGFRVIYWANLMDGELGEIKN--ITTTQP 1134

Fly           1175 DTVLHGLKKYTNYTMQVLATTAGGDGVRSVPIHCQTEPDVPEAPTDVKAL 1224
                   ...|.||:|||||::||||.|..||||||..|..:|:.|||..|..|||.
Human         1135 SLELDGLEKYTNYSIQVLAFTRAGDGVRSEQIFTRTKEDVPGPPAGVKAA 1184

Fly           1225 VMGNAAILVSWRPPAQPNGIITQYTVYSKAEGAETETKTQKVPHYQMSFE 1274
                   ....:.:.|||.||.:.||||.:|||:.........::.:..|. ..|:.
Human         1185 AASASMVFVSWLPPLKLNGIIRKYTVFCSHPYPTVISEFEASPD-SFSYR 1233

Fly           1275 ATELEKNKPYEFWVTASTTIGEGQQSKSIVAMPSDQVPAKIASFDDTFTA 1324
                   ...|.:|:.|..||.|.|:.|.|..|:.|...|..:.||:|.:|..|.|.
Human         1234 IPNLSRNRQYSVWVVAVTSAGRGNSSEIITVEPLAKAPARILTFSGTVTT 1283

Fly           1325 TFKEDAKMPCLAVGAPQPEITW--KIKGVE--FSANDRMRVLPDGSLLIK 1370
                   .:.:|..:||.|||.|.|.:.|  ...|..  .:.:.|..:.:||.:|:
Human         1284 PWMKDIVLPCKAVGDPSPAVKWMKDSNGTPSLVTIDGRRSIFSNGSFIIR 1333
```

```
Fly               1371 SVNRQDAGDYSCHAENSIAKDSITHKLIVLAPPQSPHVTLSATTTDALTV   1420
                       :|..:|:|.|||.|.|:...|.|...|.|..||..|.:|:|.||:.::|:
Human             1334 TVKAEDSGYYSCIANNNWGSDEIILNLQVQVPPDQPRLTVSKTTSSSITL   1383

Fly               1421 KLKPHEGDTAPLHGYTLHYKPEFGE-WETSEVSVDSQKHNIEGLLCGSRY   1469
                       ...|.:...:.:.||.|.|..:..| |.:..:|...:.:.:|.|.||:.|
Human             1384 SWLPGDNGGSSIRGYILQYSEDNSEQWGSFPISPSERSYRLENLKCGTWY   1433

Fly               1470 QVYATGFNNIGAGEASDILNTRTKGQKPKL-PEKPRFIEVSSNSVSLHFK   1518
                       :...|..|.:|.|..|:|:..:|.|::|:. .|:..|..:::..|.|:..
Human             1434 KFTLTAQNGVGPGRISEIIEAKTLGKEPQFSKEQELFASINTTRVRLNLI   1483

Fly               1519 AWKDGGCPMSHFVVESKKRDQIEWNQISNNVKPDNNYVVLDLEPATWYNL   1568
                       .|.|||||::.|.:|.:......|. .:.......:|::.||:.||||.|
Human             1484 GWNDGGCPITSFTLEYRPFGTTVWT-TAQRTSLSKSYILYDLQEATWYEL   1532

Fly               1569 RITAHNSAGFTVAEYDFATLTVTGGTIAPSRDLPELSAEDTIRIILSN--   1616
                       ::...||||....:.:||||...|.||.|             .|:.::.|
Human             1533 QMRVCNSAGCAEKQANFATLNYDGSTIPP-----------LIKSVVQNEE   1571

Fly               1617 -------LNLVVPVVAALLVIIIAIIVICILRSKGNHHK----------D   1649
                              |.::|.:...|:.:::.:::.::|.:.....:            .
Human             1572 GLTTNEGLKMLVTISCILVGVLLLFVLLLVVRRRRREQRLKRLRDAKSLA   1621

Fly               1650 DVVYNQTMGPGATLDKRRPDLRDELGYIAPPNRKLPPVPGSNYNTCDRIK   1699
                       :::.::......||.|::..||   .:|..|..:|            .|:
Human             1622 EMLMSKNTRTSDTLSKQQQTLR---MHIDIPRAQL------------LIE   1656

Fly               1700 RGRGGLRSNHSTWDPR-----RNPNLYEELKAPPVPM-HGNHYGHAHGNA   1743
                       .     |....|.|.|     .:.:..|..|...:.: |..||
Human             1657 E-----RDTMETIDDRSTVLLTDADFGEAAKQKSLTVTHTVHY-------   1694

Fly               1744 ECHYRHPGMEDEICPYATFHLLGF---REEMDPTKAMNFQTFP-HQNGHA   1789
                                  :....||..|:..   |...:||...|.:..| .:|.:|
Human             1695 -----------QSVSQATGPLVDVSDARPGTNPTTRRNAKAGPTARNRYA   1733

Fly               1790 G------PVP---GHAGT---MLP-PGHPGHVHSRSGSQSMPRANRYQRK   1826
                       .      |.|   .|..|   .|| |...|.|...|.|.|:..:....|.
Human             1734 SQWTLNRPHPTISAHTLTTDWRLPTPRAAGSVDKESDSYSVSPSQDTDRA   1783

Fly               1827 NSQ----GGQSSIYTPAPEYDDPANCAEEDQYRRYTRV-----NSQGGSL   1867
                       .|.    ...||.|.......:.|...|::::::|..      ::....|
Human             1784 RSSMVSTESASSTYEELARAYEHAKMEEQLRHAKFTITECFISDTSSEQL   1833

Fly               1868 YSGPGPEYDDPANCAPEEDQYGSQYGGPYGQPYDHYGSRGSMGRRSIGSA   1917
                       .:|.. ||.|....:...:....:.....:|.|    .|.:...::..|
Human             1834 TAGTN-EYTDSLTSSTPSESGICRFTASPPKPQD----GGRVMNMAVPKA   1878
```

```
Fly            1918 RNPGNGSPEPP------------PPPPRNHDMSNSSFNDSKESN------   1949
                    ..||:....||            |...|:..:..:.....|...
Human          1879 HRPGDLIHLPPYLRMDFLLNRGGPGTSRDLSLGQACLEPQKSRTLKRPTV   1928

Fly            1950 ------EISEAECDRDHGPRGNYGAVKRSPQ   1974
                          |.:.:......|.....|||...||
Human          1929 LEPIPMEAASSASSTREGQSWQPGAVATLPQ   1959


#---------------------------------------
#---------------------------------------
```
