# Supplementary material for: Tracking the evolution of alternatively spliced exons within the Dscam family
Source: BMC Evol Biol. 2006 Feb 16;6:16. doi: 10.1186/1471-2148-6-16 (PMC1397879; doi:10.1186/1471-2148-6-16)
Supplement: Additional File 7 — A Smith-Waterman alignment of fly Dscam versus Human Dscam-L, with the sequences corresponding to exons highlighted by color (color legend in the file), and putative corresponding domain locations underlined. [file 1471-2148-6-16-S7.pdf]

```
########################################
# Program: water
# Rundate: Sun Nov 18 2005 19:23:24
# Align_format: srspair
# Report_file: fly-dscamL.water
########################################

#=======================================
#
# Aligned_sequences: 2
# 1: Fly
# 2: Human
# Matrix: EBLOSUM62
# Gap_penalty: 10.0
# Extend_penalty: 0.5
#
# Length: 2181
# Identity:     633/2181 (29.0%)
# Similarity:   996/2181 (45.7%)
# Gaps:         315/2181 (14.4%)
# Score: 2523.5
#
#
#=======================================
```

fly exon 4 and human Dscam-L homolog (exon 3), with Human Dscam-L IG-like C2-type domain #2 underlined, and InterProScan predicted Fly IG PF00047.14 domain underlined

fly exon 6 and human Dscam=L homologous region (exon 5), Human Dscam-L Ig-like C2-type 3 underlined, and InterProScan fly predicted IG PF00047.14 underlined

fly exon 9.9 and human Dscam-L homolog (exon 9), with Human Dscam-L Ig-like C2-type 7 underlined, and InterProScan fly predicted IG PF00047.14 underlined

fly exon 17 and human Dscam=L homolog (exon 27), with predicted transmembrane domain underlined

```
Fly               10 WLMLFAAVALIACGSQTLAANPPDADQKGPVFLKEPTNRIDFSNSTGAEI     59
                     ||:.|..:.     .....|.|.|.. ....|:.:...::.||:|.|..:
Human             62 WLVTFLLLL-----DSLHKARPEDVG-TSLYFVNDSLQQVTFSSSVGVVV    105

Fly               60 ECKASGNPMPEIIWIRSDGTAVGDVPGLRQISSDGKLVFPPFRAEDYRQE    109
                     .|.|:|:|...:.|..:.|..:.|||.:|.:.::|.|...||....:...
Human            106 PCPAAGSPSAALRWYLATGDDIYDVPHIRHVHANGTLQLYPFSPSAFNSF    155
```

```
Fly        110 VHAQVYACLARNQFGSIISRDVHVRAVVAQYYEADVNKEHVIRGNSAVIK  159
               :|...|.|.|.|..|.|.|.::.|:||..:.|...|..:..:|||.||.|
Human      156 IHDNDYFCTAENAAGKIRSPNIRVKAVFREPYTVRVEDQRSMRGNVAVFK  205

Fly        160 CLIPSFVADFVEVVSWHTDEEENYFPGAEYDGKYLVLPSGELHIREVGPE  209
               |||||.|.::|.||||..| ..:..|    :.::.:...|.|:|.:|..|
Human      206 CLIPSSVQEYVSVVSWEKD-TVSIIP----EHRFFITYHGGLYISDVQKE  250

Fly        210 DGYKSYQCRTKHRLTGETRLSATKGRLVITEPVSSSPPKINALTYKPNIV  259
               |...:|:|.|||:.:||||.| ...||.:|:|..|.|..::.  :....|
Human      251 DALSTYRCITKHKYSGETRQS-NGARLSVTDPAESIPTILDG--FHSQEV  297

Fly        260 ESMASTAILCPAQGYPAPSFRWYKFIEGTTRKQAVVLNDR-VKQVSGTLI  308
               .:..:..:.|.|.|||.|:.||.|  :|    :.:..:.| .|:::| |.
Human      298 WAGHTVELPCTASGYPIPAIRWLK--DG----RPLPADSRWTKRITG-LT  340

Fly        309 IKDAVVEDSGKYLCVVNNSVGGESVETVLTVTAPLSAKIDPPTQTVDFGR  358
               |.|...||||.|:|.|.|:.|......:|.|..||...:.|.......|.
Human      341 ISDLRTEDSGTYICEVTNTFGSAEATGILMVIDPLHVTLTPKKLKTGIGS  390

Fly        359 PAVFTCQYTGNPIKTVSWMKDGKAI---------GHSEPVLRIESVKKED  399
               ..:.:|..||:|..|:.|.::.:.:         |.|...|.|.|.:|..
Human      391 TVILSCALTGSPEFTIRWYRNTELVLPDEAISIRGLSNETLLITSAQKSH  440

Fly        400 KGMYQCFVRNDQESAEASAELKLGGRFDPPVIRQAFQEETMEPGPSVFLK  449
               .|.||||.....::|:..|.:.|  ....|.|..:|.|:.:.||....|.
Human      441 SGAYQCFATRKAQTAQDFAIIAL--EDGTPRIVSSFSEKVVNPGEQFSLM  488

Fly        450 CVAGGNPTPEISWELDGKKIANNDRYQVGQYVTVNGDVVSYLNITSVHAN  499
               |.|.|.|.|.::|.||.:.|..:..::..||...:|..:|::|:|.....
Human      489 CAAKGAPPPTVTWALDDEPIVRDGSHRTNQYTMSDGTTISHMNVTGPQIR  538

Fly        500 DGGLYKCIAKSKVGVAEHSAKLNVYGLPYIRQMEKKAIVAGETLIVTCPV  549
               |||:|:|.|::.||.||:.|::||.|.|.||.|.....|||...::.|.|
Human      539 DGGVYRCTARNLVGSAEYQARINVRGPPSIRAMRNITAVAGRDTLINCRV  588

Fly        550 AGYPIDSIVWERDNRALPINRKQKVFPNGTLIIENVERNSDQATYTCVAK  599
               .|||..||.|.:|...||.|.:|.||.||||.:.:|::..|:..|.|...
Human      589 IGYPYYSIKWYKDALLLPDNHRQVVFENGTLKLTDVQKGMDEGEYLCSVL  638
```

```
Fly              600 NQEGYSARGSLEVQVMVPPQVLPFSFGESAADVGDIASANCVVPKGDLPL   649
                     .|...|...|:.|.|.|||.:.||.|  ..|.:|.:....|||..||:|:
Human            639 IQPQLSISQSVHVAVKVPPLIQPFEF--PPASIGQLLYIPCVVSSGDMPI   686

Fly              650 EIRWSLNSAPIVNGENGFTLVRLNKRTSLLNIDSLNAFHRGVYKCIATNP   699
                     .|.|..:...|::| :|.| :...:..|.|.|.|::..|.|.|.|||:|.
Human            687 RITWRKDGQVIISG-SGVT-IESKEFMSSLQISSVSLKHNGNYTCIASNA   734

Fly              700 AGTSEYVAELQVNVPPRWILEPTDKAFAQGSDAKVECKADGFPKPQVTWK   749
                     |.|.....:|.|.||||::::|.::.....|....:.|..||:|.|:|.||
Human            735 AATVSRERQLIVRVPPRFVVQPNNQDGIYGKAGVLNCSVDGYPPPKVMWK   784

Fly              750 KAVGD-TPGEYKDLKKSDNIRV-EEGTLHVDNIQKTNEGYYLCEAINGIG   797
                     .|.|. .|.:|..:..:..|:: ...:|.:.:.:.:.|||||:|.||:|
Human            785 HAKGSGNPQQYHPVPLTGRIQILPNSSLLIRHVLEEDIGYYLCQASNGVG   834

Fly              798 SGLSAVIMISVQAPPEFTEKLRNQTARRGEPAVLQCEAKGEKPIGILWNM   847
                     :.:|..:.::|:.|...|.......|.:|....|.|.|:||:||.|.|..
Human            835 TDISKSMFLTVKIPAMITSHPNTTIAIKGHAKELNCTARGERPIIIRWEK   884

Fly              848 NNMRLDPKNDNRYTIREEILSTGVMSSLSIKRTERSDSALFTCVATNAFG   897
                     .:..:||....||.|..:.....|:|:|.:|..:|.||..|:|.|.|::|
Human            885 GDTVIDPDRVMRYAIATKDNGDEVVSTLKLKPADRGDSVFFSCHAINSYG   934

Fly              898 SDDASINMIVQEVPEMPYALKVLDKSGRSVQLSWAQPYDGNSPLDRYIIE   947
                     .|...|.:.|||.|:.| .|::.:...||:.|.|.|.:||||.:..:.||
Human            935 EDRGLIQLTVQEPPDPP-ELEIREVKARSMNLRWTQRFDGNSIITGFDIE   983

Fly              948 FKRSRASW--SEIDRVIVPGHTTEAQVQKLSPATTYNIRIVAENAIGTSQ   995
                     :|....||  .:..|.|.| ...:|.:..|.||:.|:||:.:.|.||.|:
Human            984 YKNKSDSWDFKQSTRNISP-TINQANIVDLHPASVYSIRMYSFNKIGRSE   1032

Fly              996 SSEAVTIITAEEAPSGKPQNIKVEPVNQTTMRVTWKPPPRTEWNGEILGY   1045
                     .|:.:||.|.|.||.|.|.::.::||...::||||.|.:...||.|.||
Human           1033 PSKELTISTEEAAPDGPPMDVTLQPVTSQSIQVTWKAPKKELQNGVIRGY   1082

Fly             1046 YVGYKLSNTNSSYVFETINFITEEGKEHNLELQNLRVYTQYSVVIQAFNK   1095
                     .:||:.::..|:..:..:. :...|......|.||:.:.||.||:||||:
Human           1083 QIGYRENSPGSNGQYSIVE-MKATGDSEVYTLDNLKKFAQYGVVVQAFNR   1131

Fly             1096 IGAGPLSEEEKQFTAEGTPSQPPSDTACTTLTSQTIRVGWVSPPLESANG   1145
                     .|.||.|.|....|.|..|||||.:....::||....:.|..||..:.||
Human           1132 AGTGPSSSEINATTLEDVPSQPPENVRALSITSDVAVISWSEPPRSTLNG   1181
```

```
Fly              1146 VIKTYKVVY---APSDEWYDETKRHYKKTASSDTV-LHGLKKYTNYTMQV   1191
                      |:|.|:|::   ....||.:    ....|.:.:.| |.|::|:|||::||
Human            1182 VLKGYRVIFWSLYVDGEWGE----MQNITTTRERVELRGMEKFTNYSVQV   1227

Fly              1192 LATTAGGDGVRSVPIHCQTEPDVPEAPTDVKALVMGNAAILVSWRPPAQP   1241
                      ||.|..||||||..::.||:.|||..|..:||:....::::|||.||.:|
Human            1228 LAYTQAGDGVRSSVLYIQTKEDVPGPPAGIKAVPSSASSVVVSWLPPTKP   1277

Fly              1242 NGIITQYTVYSKAEG------AETETKTQKVPHYQMSFEATELEKNKPYE   1285
                      ||:|.:||::..:.|      :|.||..:     |:.:....|.:.:.|.
Human            1278 NGVIRKYTIFCSSPGSGQPAPSEYETSPE-----QLFYRIAHLNRGQQYL   1322

Fly              1286 FWVTASTTIGEGQQSKSIVAMPSDQVPAKIASFDDTFTATFKEDAKMPCL   1335
                      .||.|.|:.|.|..|:.:...|:.:.||||.||..|.|..:.:|.::||.
Human            1323 LWVAAVTSAGRGNSSEKVTIEPAGKAPAKIISFGGTVTTPWMKDVRLPCN   1372

Fly              1336 AVGAPQPEITW----KIKGVEFSANDRMRVLPDGSLLIKSVNRQDAGDYS   1381
                      :||.|.|.:.|    :...:..|.:....:..:|:||:::|..:|:|.|:
Human            1373 SVGDPAPAVKWTKDSEDSAIPVSMDGHRLIHTNGTLLLRAVKAEDSGYYT   1422

Fly              1382 CHAENSIAKDSITHKLIVLAPPQSPHVTLSATTTDALTVKLKPHEGDTAP   1431
                      |.|.|:...|:|...|:|..||..|.:|:|.|:..::|:...|.:...:.
Human            1423 CTATNTGGFDTIIVNLLVQVPPDQPRLTVSKTSASSITLTWIPGDNGGSS   1472

Fly              1432 LHGYTLHYKPEFG-EWETSEVSVDSQKHNIEGLLCGSRYQVYATGFNNIG   1480
                      :.|:.|.|..:.. ||:...:|...:...::.|.||:.|:|.....|::|
Human            1473 IRGFVLQYSVDNSEEWKDVFISSSERSFKLDSLKCGTWYKVKLAAKNSVG   1522

Fly              1481 AGEASDILNTRTKGQKPKL-PEKPRFIEVSSNSVSLHFKAWKDGGCPMSH   1529
                      :|..|:|:..:|.|::|.. .::..|..::|....|:.:.|.:||||::.
Human            1523 SGRISEIIEAKTHGREPSFSKDQHLFTHINSTHARLNLQGWNNGGCPITA   1572

Fly              1530 FVVESKKRDQIEWNQISNNVKPDNNYVVLDLEPATWYNLRITAHNSAGFT   1579
                      .|:|.:.:....|..:..|  ......:.:|..||||.||:.|.||||..
Human            1573 IVLEYRPKGTWAWQGLRAN--SSGEVFLTELREATWYELRMRACNSAGCG   1620

Fly              1580 VAEYDFATLTVTGGTIAPSRDLPELSAE---DTIRIILSNLNLVVPVVAA   1626
                      .....||||...|.||.|.:     ||:   |.::.:.:   :..||:.|
Human            1621 NETAQFATLDYDGSTIPPIK-----SAQGEGDDVKKLFT---IGCPVILA   1662
```

```
Fly                1627 LLVIIIAIIV------------------ICILRSKGNHHKDDVVYNQTMG   1658
                        .|.:.:..||                  ..:|.||.|...|..|.....|
Human              1663 TLGVALLFIVRKKRKEKRLKRLRDAKSLAEMLISKNNRSFDTPVKGPPQG   1712

Fly                1659 PGATLDKRRPDL----RDELGYIAPPNRKLP-------------------   1685
                        |...:|..|..|    ::.:.:.....:|
Human              1713 PRLHIDIPRVQLLIEDKEGIKQLGDDKATIPVTDAEFSQAVNPQSFCTGV   1762

Fly                1686 ------------PV-------PGSNYNTCDRIKRGRGGLRSNHST-----   1711
                                    |:       ||:|       ...|..::|.|||
Human              1763 SLHHPTLIQSTGPLIDMSDIRPGTN-------PVSRKNVKSAHSTRNRYS   1805

Fly                1712 --------------------W-------------------------DPRR   1716
                                            |                         |..|
Human              1806 SQWTLTKCQASTPARTLTSDWRTVGSQHGVTVTESDSYSASLSQDTDKGR   1855

Fly                1717 N--------PNLYEELKAPPVPMHGNHYGHAHGNAECHYRHPGMEDEICP   1758
                        |        .:.||||...                   |.|..:|::: .
Human              1856 NSMVSTESASSTYEELARA-------------------YEHAKLEEQL-Q   1885

Fly                1759 YATFHL--------------LGFREEMDPTKAMNFQTFPHQNGHAGPVPG   1794
                        :|.|.:              .|..|..|...:|:             .|.
Human              1886 HAKFEITECFISDSSSDQMTTGTNENADSMTSMS-------------TPS   1922

Fly                1795 HAG----TMLPPGHPGHVHSRSGSQSMP-RANR--------YQRKNSQGG   1831
                        ..|    |..||........::.:..:| |||:        |.:.:.:...
Human              1923 EPGICRFTASPPKPQDADRGKNVAVPIPHRANKSDYCNLPLYAKSEAFFR   1972

Fly                1832 QSSIYTPAPEYDDPANCAEEDQYRRYTRVNSQGGSLYSGPGPEYDDPANC   1881
                        ::....|.|.. .|...:.:..|.|   ::|...|..       |||:
Human              1973 KADGREPCPVV-PPREASIRNLARTY---HTQARHLTL-------DPAS-   2010

Fly                1882 APEEDQYGSQYGGPY-GQPYDHYGSRGSMGRRSIGSARNPGNGSPEPPPP   1930
                                ...|.|: |.|  ...|..::.:|::.....|...:|..|.|
Human              2011 --------KSLGLPHPGAP--AAASTATLPQRTLAMPAPPAGTAPPAPGP   2050

Fly                1931 PPRNHDMSNSSFNDSKESNEISEAECDRDHGPRGNYGAVKRSPQPKDQRT   1980
                        .|.....:.|:      :......|..|..||....|..:.|
Human              2051 TPAEPPTAPSA------APPAPSTEPPRAGGPHTKMGGSRDS--------   2086

Fly                1981 TEEMRKLIERNETGPKQLQLQQANGAGFTAY   2011
                              |:|.:.:|..:.|.|   |||  ||
Human              2087 ------LLEMSTSGVGRSQKQ---GAG--AY   2106
```
